# First record of the genus *Phradis* Förster (Hymenoptera, Ichneumonidae, Tersilochinae) from the Neotropical Region

**DOI:** 10.3897/zookeys.169.2333

**Published:** 2012-02-10

**Authors:** Andrey I. Khalaim, Santiago Bordera

**Affiliations:** 1 Zoological Institute, Russian Academy of Sciences, Universitetskaya nab. 1, St. Petersburg 199034, Russia. División de Estudios de Postgrado e Investigación, Facultad de Ingeniería y Ciencias, Universidad Autónoma de Tamaulipas, Cd. Victoria 87149, México; 2 Instituto de Investigación de Biodiversidad CIBIO, Universidad de Alicante, Ap. Corr. 99, 03080-Alicante, Spain

**Keywords:** Peru, Western Amazonia, *Phradis*, new species, taxonomy

## Abstract

One new species of the genus *Phradis*, *Phradis peruvianus* **sp. n.**, from the mountainous part of Peruvian Amazonia, is described and illustrated. This is the first record of the genus from South America and the Neotropical region.

## Introduction

*Phradis* Förster, 1869 is one of the most species rich genera of Tersilochinae with almost world wide distribution (not recorded from the Neotropical and Oriental regions and absent in New Zealand). The genus is best represented in the Holarctic region and comprises 38 species in the Palaearctic region (Khalaim 2007b, Khalaim et al. 2009), and one described (Khalaim 2002) and 17 undescribed species in the Nearctic region (Horstmann in press.); all these species are restricted either by Palaearctic or Nearctic region. Very few taxa are known beyond the Holarctic region; two species were described from South Africa (Khalaim 2007a), and five undescribed species were mentioned from Australia by Gauld (1984).

The genus belongs to the “*Phradis*” group of genera as well as the genera *Allophrys* Förster and *Heterocola* Förster (Horstmann 1981). Genera of this group can easily be distinguished by the combination of first metasomal segment without glymmae, propodeum with basal area or rarely with basal groove, without basal keel, and also by fore wing usually with second recurrent vein interstitial or antefurcal, mesopleuron usually without or with weak and short foveate groove and propodeal spiracle separated from pleural carina by 3–5 diameters of spiracle (this distance is much shorter in most other tersilochines). Within this group of genera, the genus *Phradis* differs from *Heterocola* by short maxillary and labial palpi (extremely long in *Heterocola*), interstitial (rarely antefurcal or slightly postfurcal) second recurrent vein (always strongly antefurcal in *Heterocola*), usually longer basal area of propodeum, and larger distance between propodeal spiracle and pleural carina. It also differs from *Allophrys* by nervellus less reclivous, eyes of both sexes not enlarged, with inner margins more or less parallel, being conspicuously enlarged and strongly convergent dorsad in males of *Allophrys* (females of *Allophrys* possess not enlarged eyes similar to that in *Phradis*, thus sometimes females of these two genera are hardly distinguishable), and usually lacking hypostomal carina (well developed in *Allophrys*).

Some species of *Phradis* are common parasitoids of sap beetle larvae (Coleoptera: Nitidulidae: *Meligethes* spp.) on rape in Europe (Horstmann 1971, 1981, Nilsson 1985, Nilsson and Andreasson 1987, Osborne 1960, etc).

Studying large quantities of material of Tersilochinae from Western Amazonia, a new species of the genus has been found from the mountainous Peruvian Amazonia.

## Material and methods

About 530 specimens of Tersilochinae from the ichneumonid collections of the Zoological Museum of University of Turku, Finland, the Entomological Museum Klaus Raven Büller of Universidad Nacional Agraria la Molina, Lima, Peru, and the Entomological Collection of the University of Alicante, Spain, collected in Western Amazonia (Ecuador and Peru) in 1994–2010, have been studied. Material of *Phradis* was collected in Poromate basin (Quillabamba, La Convención Prov., Cusco Dept., Peru), a premontane area at 1,600 m a.s.l., mostly covered with a closed to open broadleaved evergreen or semi-deciduous subtropical dry forest. The climate is classified as dry sub-humid (dry winter, warm summer). The soil in the area is high in leptosols with a weakly developed shallow soil.

Morphological terminology predominantly follows Townes (1969) with changes according to Khalaim (2011).

The type material of the new species described in the present paper is deposited in the Museo de Entomología Klaus Raven Büller (MEKRB), in the Entomological Collection of University of Alicante (CEUA), and in the Zoological Institute of Russian Academy of Sciences, St. Petersburg, Russia (ZISP).

Layer photos were taken in the Zoological Museum of University of Turku, Finland, using an Olympus SZX16 stereomicroscope attached to an Olympus E520 digital camera. Digital photos were combined by using the software Deep Focus 3.1. Scanning electron microscope images were taken in the University of Alicante using a Hitachi S-3000N in low vacuum mode. All images were assembled and edited with Adobe Photoshop CS2 software.

## Results

### Phradis peruvianus

Khalaim & Bordera sp. n.

urn:lsid:zoobank.org:act:98014132-8082-4A52-ADFF-E44B5B1EEDB6

http://species-id.net/wiki/Phradis_peruvianus

Figs 1 –10

#### Diagnosis.

 Differs from other species of the genus by the extremely deep and broad foveate groove of mesopleuron extending from anterior margin of mesopleuron to base of mid coxa (Fig. 9). It is also characterized by the slender, black, 17-segmented flagellum (Fig. 6), propodeum with broad, impressed, longitudinally wrinkled basal area (basal longitudinal carinae indistinct), and fore wing with interstitial or slightly antefurcal second recurrent vein.

**Figures 1–2.**
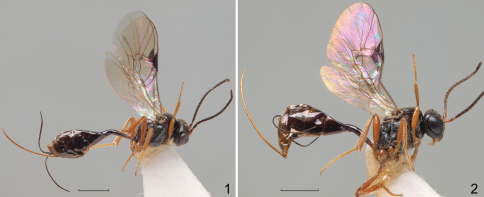
*Phradis peruvianus* sp. n. **1** holotype **2** paratype.<br/>

#### Description.

 Female. Body length 4.6 mm. Fore wing length 3.8 mm.

**Figures 3–10.**
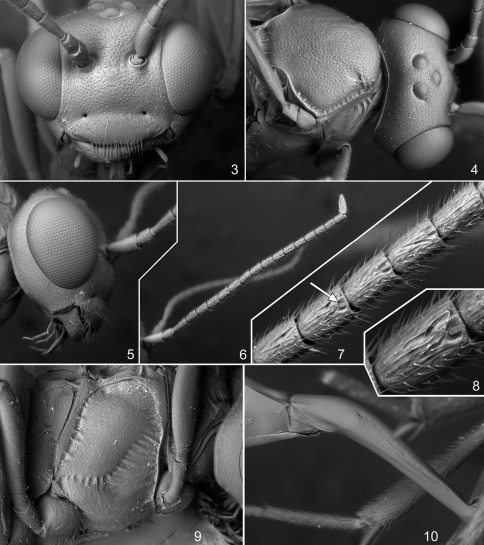
*Phradis peruvianus* sp. n. **3** head, frontal view **4** head and mesoscutum, dorsal view **5** head, lateral view **6** antenna, lateral view **7** flagellomeres **3–7** of antenna, lateral view **8** flagellomere 4 of antenna, lateral view **9** mesopleuron, lateral view **10** base of metasoma, lateral view.

**Head.** Roundly narrowed behind eyes in dorsal view; temple 0.42–0.48 times as long as eye width (Fig. 4). Distance between lateral ocellus and eye 2.2 times the maximum diameter of ocellus. Upper tooth of mandible much longer and broader than lower tooth. Clypeus lenticular, mostly smooth, finely punctate near its upper margin, with few larger punctures in its upper half and apical margin with a row of long setae (Fig. 3). Malar space 0.6 times as long as basal width of mandible. Flagellum of antenna with 17 flagellomeres (Fig. 6); second flagellomere almost twice as long as broad, subapical flagellomere about 1.6 times as long as broad; flagellomeres 4–6 bearing finger-shaped structures near apex on outer surface (Figs 7, 8). Inner eye orbits slightly convergent ventrally. Face and frons densely punctate on a granulate background. Vertex matt, finely punctate-granulate. Temple finely punctate, more or less smooth between punctures. Occipital carina complete.

**Mesosoma.** Notaulus short, rather deep, with strong wrinkle (Fig. 4). Mesoscutum densely punctate on a granulate background. Scutellum with lateral longitudinal carinae reaching from its base to about posterior 0.5. Foveate groove of mesopleuron extending from anterior margin of mesopleuron to base of mid coxa, deep, anteriorly very broad and with strong transverse wrinkles, posteriorly narrow and crenulate (Fig. 9). Mesopleuron predominantly smooth, with fine, moderately dense punctures. Propodeum granulate, dull, dorsolateral area very finely punctate, apical area with more or less distinct fine transverse wrinkles. Basal part of propodeum short, 0.25–0.27 times as long as apical area. Basal longitudinal carinae indistinct, propodeum dorsally with broad impressed area with few longitudinal wrinkles. Propodeal spiracle very small, round; distance between spiracle and pleural carina equal to about 3.0 diameters of spiracle. Apical area flat, anteriorly broadly rounded, sometimes slightly truncated.

**Wings.** Fore wing with second recurrent vein interstitial or slightly antefurcal, unpigmented in its anterior about 0.4. Intercubitus rather long. First abscissa of radius straight, distinctly longer than width of pterostigma. Metacarpus almost reaching apex of fore wing. Hind wing with nervellus slightly inclivous.

**Legs.** Hind femur 4.6 times as long as broad, and 0.82–0.84 times as long as tibia. Hind spurs almost straight, slightly curved at apex. Tarsal claws not pectinate.

**Metasoma.** First tergite slender, 4.7 times as long as posteriorly broad, smooth and shiny; down-curved from level of spiracle to the end of postpetiole, with some striae laterally, round in transverse cross-section (Fig. 10). Glymma absent. Second tergite 1.9–2.0 times as long as anteriorly broad. Thyridial depression about twice as long as broad. Ovipositor upcurved, thin, with shallow, dorsal, subapical depression; sheath about 1.8 times as long as first tergite and 1.85–1.92 times as long as hind tibia.

**Coloration.** Head, mesosoma (including tegula) and flagellum of antenna black; clypeus slightly brownish in its lower part; palpi, mandible (teeth blackish), scape and pedicel of antenna and legs reddish brown. Pterostigma and metasoma dark brown to brownish black.

Male unknown.

#### Variation.

 All specimens are very similar in structure and colour, without obvious variation.

#### Material examined.

Holotype female, Peru, Cuzco Prov., La Convención, Quillabamba, Poromate, 1600 m, 12.XII.2007, leg. A. Rodríguez (MEKRB). Paratypes: one female with same data as holotype (CEUA), one female with same data except 11.XI.2007 (ZISP).

#### Distribution.

 Peru (Cuzco).

#### Etymology.

 The species name means “from Peru”.

## Discussion

Very little is known about Tersilochinae of the Neotropical Region. Only eight species of *Stethantyx* Townes (Blanchard 1945, Graf 1980), two species of *Allophrys* Förster (Horstmann 2010) and one species of *Meggoleus* Townes (Townes 1971) were described from this region. *Stethantyx* is a dominant tersilochine genus in the Neotropical region with at least 20 undescribed species in Costa Rica and 14 in Western Amazonia (Khalaim pers. obs.), in addition to eight previously described species which occur in south-eastern and southern Brazil, Argentina and Uruguay. *Meggoleus spirator* Townes is widely distributed through the Neotropical region, extending from Guatemala to Southern Brazil (Khalaim and Broad in press). Among two described species of *Allophrys*, *Allophrys oculata* (Ashmead) is known only from Grenada in West Indies, and another one, the recently described *Allophrys divaricata* Horstmann, is widely distributed from south-eastern U.S.A. to Argentina (Horstmann 2010). Over ten undescribed species of *Allophrys* also occur in Costa Rica (Khalaim and Broad in press) and about six species are recognized in Western Amazonian material (Khalaim pers. obs.).

The genus *Phradis* has almost a world wide distribution but is not recorded from the Neotropical and Oriental regions and is also absent in New Zealand (Khalaim pers. obs.), although it is quite possible that representatives of this genus may be found in the Oriental region, as some other large Palaearctic tersilochine genera (*Barycnemis* Förster, *Probles* Förster and *Tersilochus* Holmgren) are represented by one or few species in this region (Khalaim 2011).

In North America only the species *Phradis kasparyani* Khalaim was described from California, U.S.A. (Khalaim 2002), but neither this nor other species of *Phradis* were recorded from Mexico, where over 100 specimens of Tersilochinae from Tamaulipas and some other Mexican provinces were studied by the first author. A large number of tersilochines was also collected during many years in various terrestrial biotopes of Costa Rica (Gauld 1991 and further publications), but no species of *Phradis* was registered from this country (Khalaim pers. obs.). This can suggest that this genus does not occur in Costa Rica, and probably nowhere in Central America. But a single species of *Phradis* has been found in Western Amazonian material, where only three specimens were collected in a mountainous area of Peruvian Amazonia. This is the first and unexpected finding of *Phradis* in tropical America.

Regarding the morphology of the new species we can highlight the possession, near the apex of the outer surface of flagellomeres 4–6, of finger-shaped structures. Such structures also were recently described in two European species of *Phradis* (Khalaim et al. 2009) and later in many Costa Rican species of other tersilochines (in all species of *Allophrys*, in one undescribed species of *Barycnemis* and in *Meggoleus spirator*; Khalaim and Broad in press). We have seen this also in one species of *Barycnemis* in Europe (Bordera pers. obs.), and in one undescribed species of *Stethantyx* from Argentina (Khalaim pers. obs.). These structures are very small and hardly visible in light microscope, but very distinct in SEM photos (Figs 7, 8). These observations may suggest that these structures may occur in many tersilochine genera, but they have not been registered before because of their small size. The structures have been observed both in males and females and their function could be sensitive sensilla but further studies should demonstrate their real function.

## Supplementary Material

XML Treatment for Phradis peruvianus
